# Use of a Green Familiar Faces Paradigm Improves P300-Speller Brain-Computer Interface Performance

**DOI:** 10.1371/journal.pone.0130325

**Published:** 2015-06-18

**Authors:** Qi Li, Shuai Liu, Jian Li, Ou Bai

**Affiliations:** 1 School of Computer Science and Technology, Changchun University of Science and Technology, Changchun, China; 2 Hequ Hydrologic Station, Middle Reaches Hydrology and Water Resources Bureau of Yellow River Conservancy Commission, Jinzhong, China; 3 School of Engineering, Virginia Commonwealth University, Richmond, United States of America; UCLA, UNITED STATES

## Abstract

**Background:**

A recent study showed improved performance of the P300-speller when the flashing row or column was overlaid with translucent pictures of familiar faces (FF spelling paradigm). However, the performance of the P300-speller is not yet satisfactory due to its low classification accuracy and information transfer rate.

**Objective:**

To investigate whether P300-speller performance is further improved when the chromatic property and the FF spelling paradigm are combined.

**Methods:**

We proposed a new spelling paradigm in which the flashing row or column is overlaid with translucent green pictures of familiar faces (GFF spelling paradigm). We analyzed the ERP waveforms elicited by the FF and proposed GFF spelling paradigms and compared P300-speller performance between the two paradigms.

**Results:**

Significant differences in the amplitudes of four ERP components (N170, VPP, P300, and P600f) were observed between both spelling paradigms. Compared to the FF spelling paradigm, the GFF spelling paradigm elicited ERP waveforms of higher amplitudes and resulted in improved P300-speller performance.

**Conclusions:**

Combining the chromatic property (green color) and the FF spelling paradigm led to better classification accuracy and an increased information transfer rate. These findings demonstrate a promising new approach for improving the performance of the P300-speller.

## Introduction

The brain-computer interface (BCI) provides an alternative communication channel that is independent of muscular control [1–4]. Non-invasive BCI systems commonly utilize electroencephalography (EEG) signals from the scalp to control external computers or machines, which have been found to be particularly useful for patients with amyotrophic lateral sclerosis (ALS) and locked-in state (LIS) [5–6].

The P300-speller system is one of the most commonly used non-invasive BCI systems; its name derives from the fact that it mainly relies on P300 and other event-related potentials (ERPs) [7–8]. Through the P300-speller system, users can communicate a specific character by attending to the cell of the matrix that contains the desired character (target character) and counting the number of times it is intensified (or flashed) [9]. However, performance of the P300-speller BCI system is not yet satisfactory due to its low classification accuracy and information transfer rate (ITR) [10].

A large amount of research has been conducted to improve the performance of the P300-speller system by optimizing the signal-processing algorithm and developing novel classification techniques, such as step-wise discriminant analysis, wavelets, and support vector machines [11–13]. In addition, some researchers have tried to improve the performance of this system by optimizing the physical properties of the spelling paradigm, such as the matrix size [14], the stimulation frequency [15], the inter-stimulus interval (ISI) [9, 15], the stimulation intensity [16], and other factors [17–18].

Recent research has focused on the manipulation of spelling paradigm stimuli to increase other ERP components that occur before or after the P300 potential for the purpose of enhancing the difference between the attended and ignored characters [19]. For example, Kaufmann et al. (2011) superimposed a row or column of the P300-speller with translucent pictures of familiar faces (Albert Einstein or Ernesto “Che” Guevara) (FF spelling paradigm) and found its performance was markedly superior to the conventional P300-speller system and the 'flash only' P300-speller system, as it evoked additional N170 and N400f ERPs [10, 20]. Some studies showed that the N170 occurs between 130 and 200 ms post-stimulus and has been associated with the pre-categorical structural encoding of faces [21–22]. The N400f occurs between 300 and 500 ms post-stimulus at the parietal and central electrode sites, and is related to the familiarity component of face recognition [19, 23]. As this technology advanced, many researchers attempted to further optimize the FF spelling paradigm to improve the performance of the P300-speller system. For example, a facial expression changes paradigm was developed to decrease adjacent interference [24], and a multi-faces paradigm was used to decrease repetition [8]. The FF spelling paradigm was also tested in patients and demonstrated good performance [25]. In addition, Takano et al. (2009) found that the color of the stimuli could also influence P300-speller system performance. They replaced the white/gray flicker matrix with a green/blue flicker matrix and found that this chromatic stimulus improved the performance of the P300-speller system [26]. Therefore, we hypothesized that combining the chromatic property and the FF spelling paradigm may lead to a better classification and ITR.

In the present study, we proposed a new spelling paradigm in which the flashing row or column is overlaid with translucent green pictures of familiar faces (GFF spelling paradigm). We analyzed the elicited ERP waveforms induced by the FF spelling paradigm and by the proposed GFF spelling paradigm, and compared P300-speller BCI system performance between the two spelling paradigms.

## Methods

### Participants

**Fig 1:**
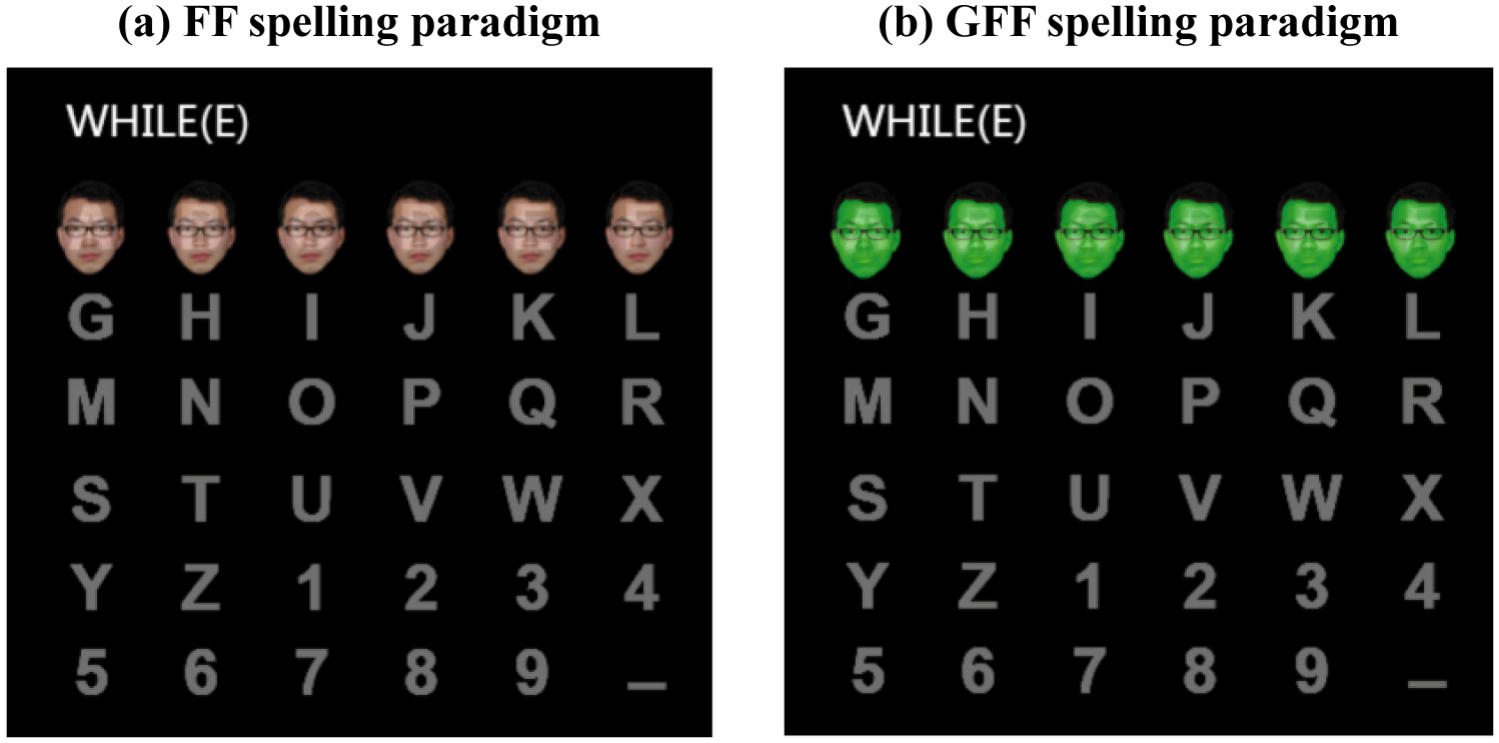
Two different spelling paradigms were designed and employed in this study. Translucent pictures of a familiar face (David Beckham) covered the characters in one row or column while it was intensified. (a) In the FF spelling paradigm, the characters were covered with flashing familiar faces. (b) In the GFF spelling paradigm, the characters were covered with flashing green familiar faces. The facial photographs of David Beckham are replaced by those of a subject in the figure because of the lack of a print license. The individual in the photograph has given permission for his photograph to be published.

The study comprised one offline and one online experiment. Seventeen university students (6 female students; age, 21–26 years; mean age: 24.6 years) participated in the offline experiment, and 12 of these also participated in the online experiment. The participants did not have any known neurological disorders, and had normal or corrected-to-normal vision. After receiving a full explanation of the purpose and risks of the study, participants signed a written informed consent and were paid 50 RMB per experiment. The individual whose facial photograph is shown in Fig 1 provided written informed consent (as outlined in PLOS consent form) for publication of the photograph. The study was approved by the ethics committee of Changchun University of Science and Technology (CUST). All participants were native Chinese speakers, but were familiar with the Western characters used in the display.

### The spelling paradigms

We designed two P300-speller spelling paradigms based on the conventional P300-speller spelling paradigm. In each paradigm, 36 spelling characters were presented in a 6 × 6 matrix subtended 13.4°×19.4° (24 × 16.5 cm) visual angle on a 19-in screen at a refresh rate of 60 Hz (Fig 1). The size of each character was 1.2° × 1.2° (1.5 × 1.5 cm). The distance between each character in was 3.7° × 2.5° (4.5 × 3 cm). The rows and columns of the matrix were flashed consecutively in pseudorandom order. In the first paradigm, the rows or columns of the characters were covered with translucent pictures of a familiar face (David Beckham) while they were flashed (FF spelling paradigm, Fig 1a). The ISI was set to 250 ms, in which each character was changed to the face picture for 200 ms, and then reverted to gray characters for 50 ms. The second spelling paradigm was similar to the first, but the translucent pictures of the familiar faces were painted green (GFF spelling paradigm, Fig 1b). We used the same brightness (20 cd/cm) and contrast to prevent these parameters from affecting the results.

### Procedure

**Fig 2:**
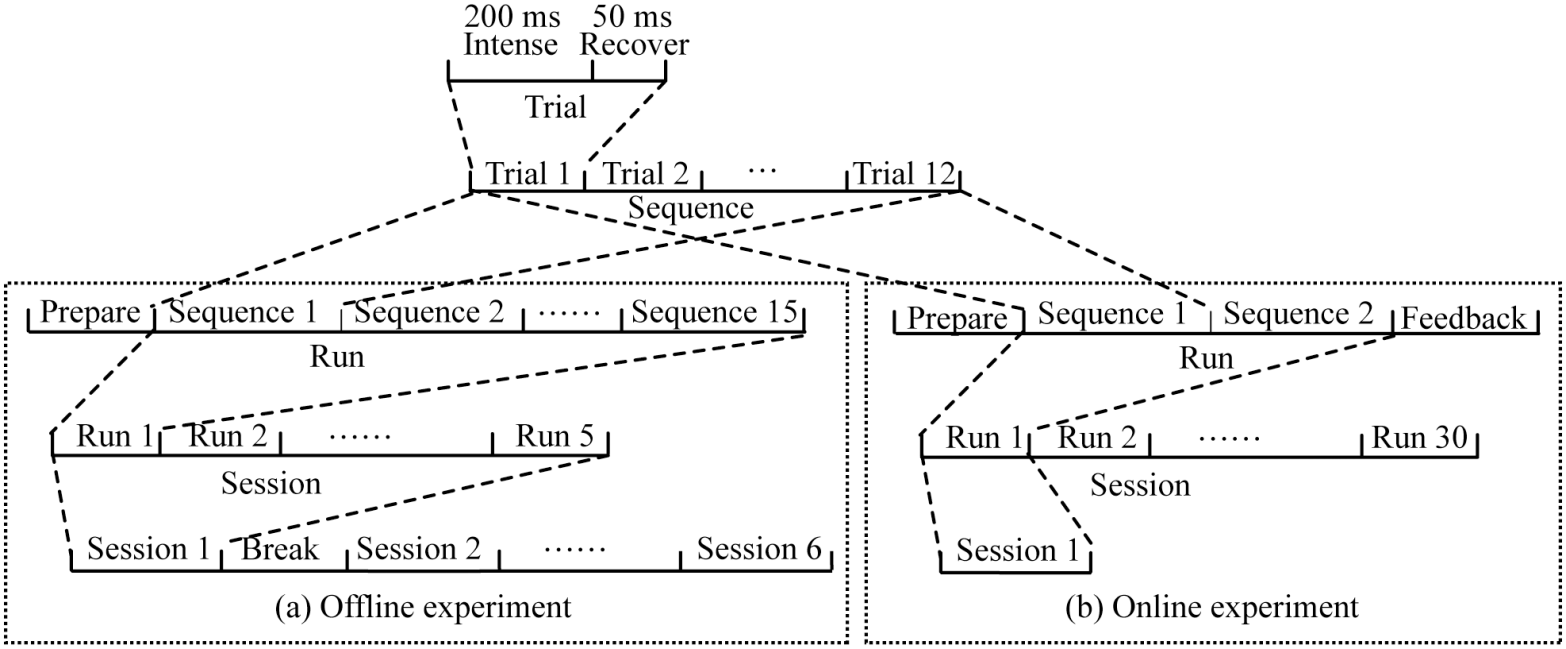
Experimental arrangement of each spelling paradigm in the offline (a) and online (b) experiments.

Each subject sat in a comfortable chair approximately 70 cm from the front of the computer monitor. Subjects were asked to focus on the target character, avoid blinking during stimulus presentation, and silently count the number of target character flashes. In the offline experiment, each spelling paradigm was conducted six times with different five-character words, and each was considered a separate session. Each session consisted of five runs, each of which involved a different target character. One flash of a row or column was referred to as a trial. The flash of a row or column including the target character was defined as a target trial, and the flash of a row or column without the target character was defined as a non-target trial. A sequence consisted of 12 flashes (trails), six from the rows and six from the columns. In each run, the sequence was repeated 15 times (Fig 2a). Thus, each run consisted of 180 flashes of row or column to output a target character. Participants never received feedback. The sessions of the two paradigms were conducted alternately to control for potential habituation effects. Participants were allowed to take a 5-min break between sessions. To avoid novelty effects, the stimulus image in each spelling paradigm was presented to subjects for 20 s prior to each session.

The online experiment was implemented in a different day. Each spelling paradigm was conducted one time as a separate session. Each session consisted of 30 runs, each of which involved a different target character. The number of sequences per run was two. Feedback regarding spelling correctness was provided to the subjects after each of the runs (Fig 2b).

### Data acquisition

**Fig 3:**
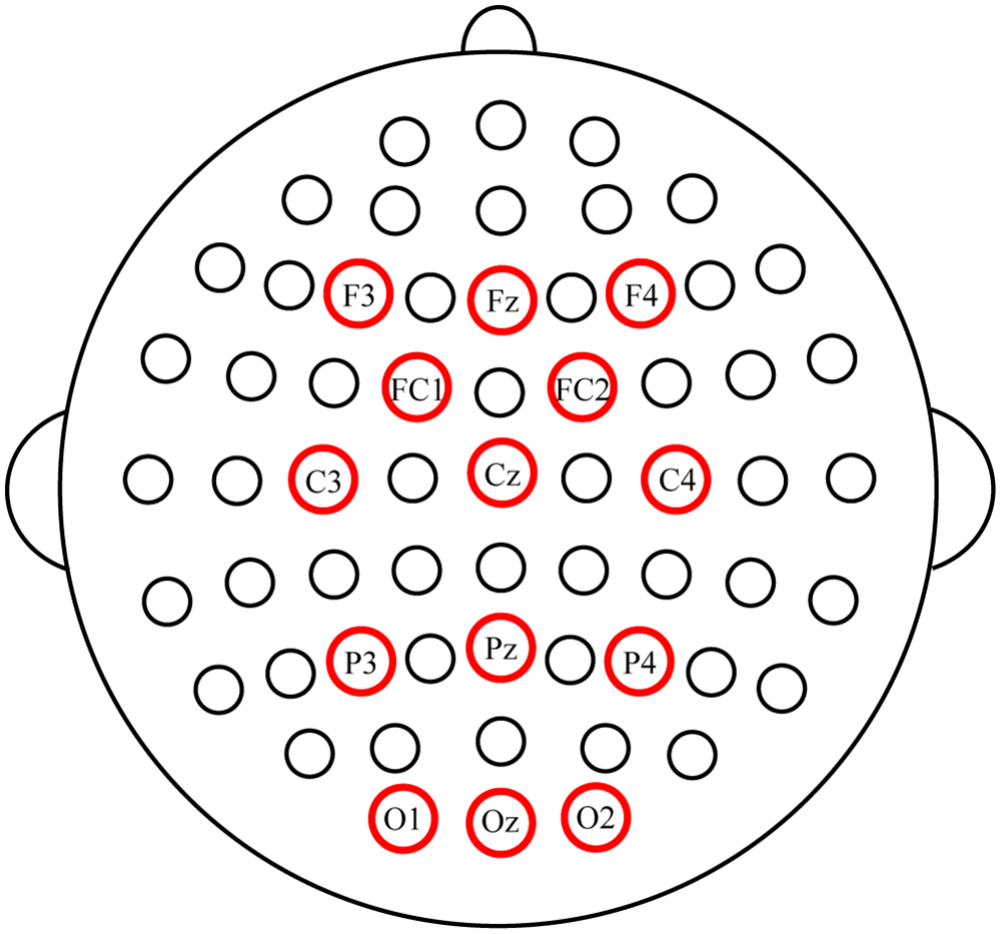
EEG setup consisting of 14 electrodes. Locations were Fz, F3, F4, FC1, FC2, Cz, C3, C4, Pz, P3, P4, Oz, O1, and O2.

Fourteen-channel (Fz, F3, F4, FC1, FC2, Cz, C3, C4, Pz, P3, P4, Oz, O1, and O2; Fig 3) electroencephalogram (EEG) data were recorded with the left mastoid as the ground and the right mastoid as the reference. Horizontal eye movements were measured by deriving the electrooculogram (EOG) from a pair of horizontal EOG (HEOG) electrodes placed at the outer canthi of the left and right eyes. Vertical eye movements and eye blinks were detected by deriving an EOG signal from a pair of vertical EOG (VEOG) electrodes placed approximately one centimeter above and below the subject’s left eye. The impedance was maintained below 5 kΩ. All signals were band-pass filtered at 0.1–100 Hz, amplified with a NeuroScan amplifier (SynAmps 2, NeuroScan Inc., Abbotsford, Australia), and digitized at a rate of 250 Hz. Stimulus presentation was controlled by a personal computer running Presentation 0.71 software (Neurobehavioral Systems Inc., Albany, NY, USA). Data acquisition was conducted using Scan4.5 software (NeuroScan Inc.).

### Event-related potentials (ERP) processing

Before the offline classification, we compared the difference waveforms of ERPs elicited by the target and non-target trials in the FF spelling paradigm with those of the GFF spelling paradigm.

EEG data were digitally filtered using a band-pass filter of 0.01–30 Hz and were corrected for ocular artifacts using a regression analysis algorithm [27]. EEG signals were divided into epochs from 100 ms before the onset of each trial to 800 ms after the onset, and baseline corrections were made against -100–0 ms. The ERP data were averaged for each trial type (target, non-target trials). The grand-averaged ERP data were obtained from all participants for each trial type in the two spelling paradigms. The difference waveform (ERPTarget—ERPNon-target) was computed by subtracting ERP waveforms elicited by non-target trials from those elicited by target trails in both FF and GFF spelling paradigms.

The mean amplitudes were calculated for all electrodes at consecutive 20 ms windows between stimulus onset and 800 ms after stimulus presentation, and the data were then analyzed using ANOVA with the within-subjects factors of spelling paradigm (FF, GFF spelling paradigm), time window (40 levels), and electrodes (14 levels). The Greenhouse–Geisser Epsilon correction was applied to adjust the degrees of freedom of the F ratios, if necessary. In order to determine the electrodes and time periods in which there was a significant difference between the two spelling paradigms, a multiple comparison was conducted with the within-subjects factors of 2 spelling paradigms (FF and GFF spelling paradigms) × 40 time windows × 14 electrodes. All statistical analyses were conducted using the SPSS version 19.0 software package (SPSS Inc., Chicago, IL, USA).

### Classification scheme

Bayesian linear discriminant analysis (BLDA) was used to classify the EEG data. BLDA is an extension of Fisher's linear discriminant analysis (FLDA) that avoids over fitting, which has be demonstrated to obtain very good classification performance in familiar faces P300-speller BCI applications [8, 19, 24]. The details of the algorithm can be found in [28]. We used six-fold cross-validation to calculate the individual accuracy in the offline experiment (i.e., we sequentially chose one of the six sessions as the test session and obtained six different training and test session groups; the accuracy of each of the six groups was computed; the individual accuracy of each participant was obtained by averaging the six results). Data acquired offline were used to train the classifier using BLDA and obtain the classifier model. This model was then used in the online experiment. If there was a tie between multiple characters, the classifier would automatically select the last output as the target character.

### Information transfer evaluation

Information transfer rate (ITR) is generally used to evaluate the communication performance of a BCI system and is a standard measure that accounts for accuracy, the number of possible selections, and the time required to make each selection [4, 20, 29]. For a sequence with N possible choices in which each choice has an equal probability of selection by the user, the probability (P) that the desired choice will indeed be selected remains invariant, and each error choice has the same probability of selection, the ITR (bits min-1) can be calculated as

$$ITR=M\left\{{\text{log}}_{2}^{N}+P{\text{log}}_{2}^{P}+(1-P){\text{log}}_{2}^{\left(\frac{1-P}{N-1}\right)}\right\}$$ (1)

where M denotes the number of commands per minute.

## Results

### ERP results

**Fig 4:**
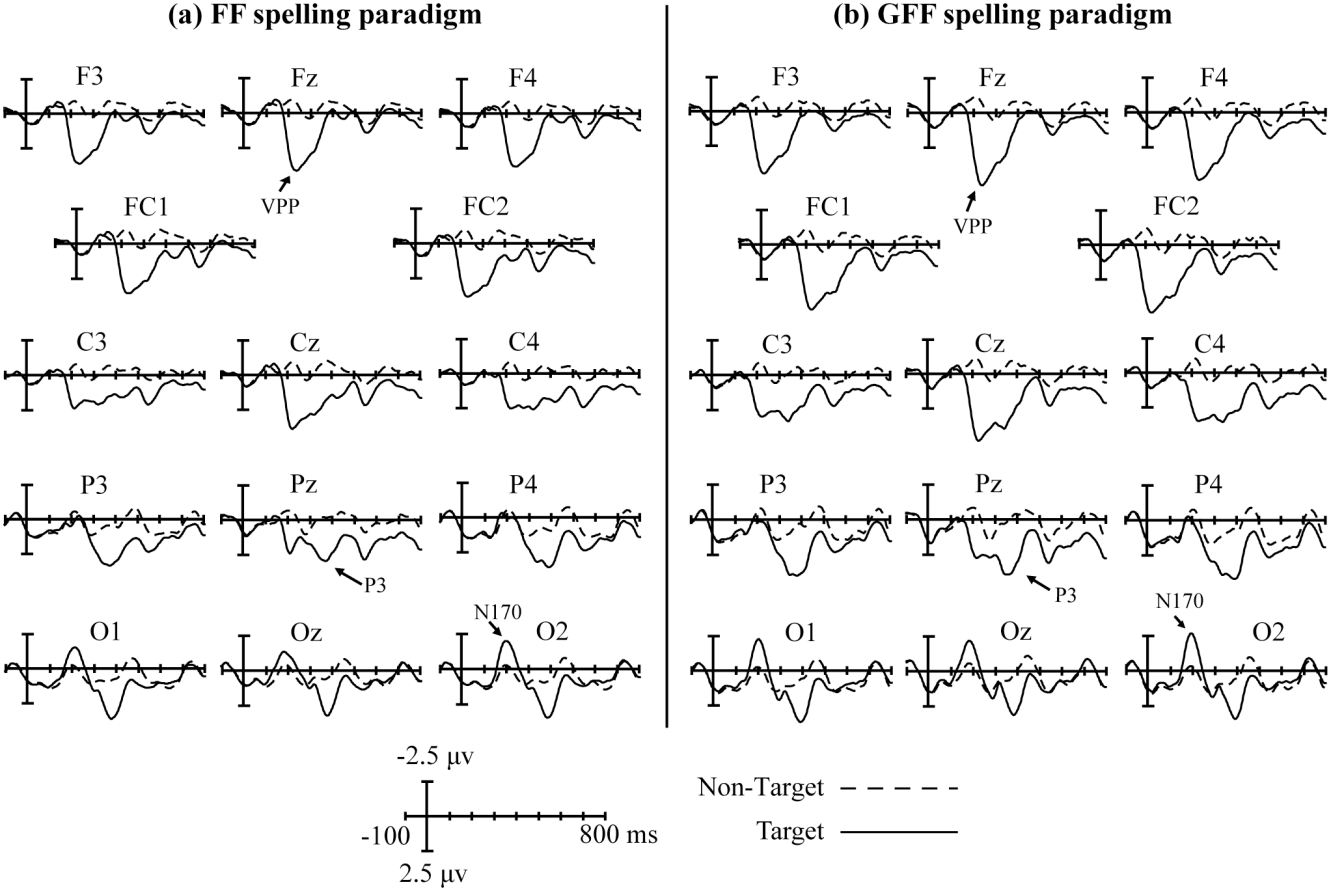
Superimposed grand-averaged ERP waveforms elicited by non-target and target trials in the FF and GFF spelling paradigms. The epochs are from 100 ms before stimulus onset to 800 ms after onset.

Fig 4 displays the superimposition of grand-averaged ERP waveforms elicited by non-target trials and target trials in the FF and GFF spelling paradigms.

In both paradigms, a negative ERP component was observed between 150 and 250 ms at the temporal occipital area for target trials, and its amplitude peaked at the O2 electrode at around 196 ms (-1.974μV) in the FF spelling paradigm, and at around 192 ms (-2.659 μV) in the GFF spelling paradigm. The distribution was slightly asymmetric insofar as the right was larger than the left. A positive ERP component was observed between 180 and 380 ms in the frontal area for target trials, and its amplitude peaked at the Fz electrode at around 236 ms (4.185 μV) and 232 ms (5.263 μV) in the FF and GFF spelling paradigm, respectively. The third ERP component was found between 300 and 450 ms for target trials and was a positive ERP component. The amplitude peaked at the Pz electrode at around 372 ms (2.951 μV) in the FF spelling paradigm and at around 352 ms (3.858 μV) in the GFF spelling paradigm.

**Fig 5:**
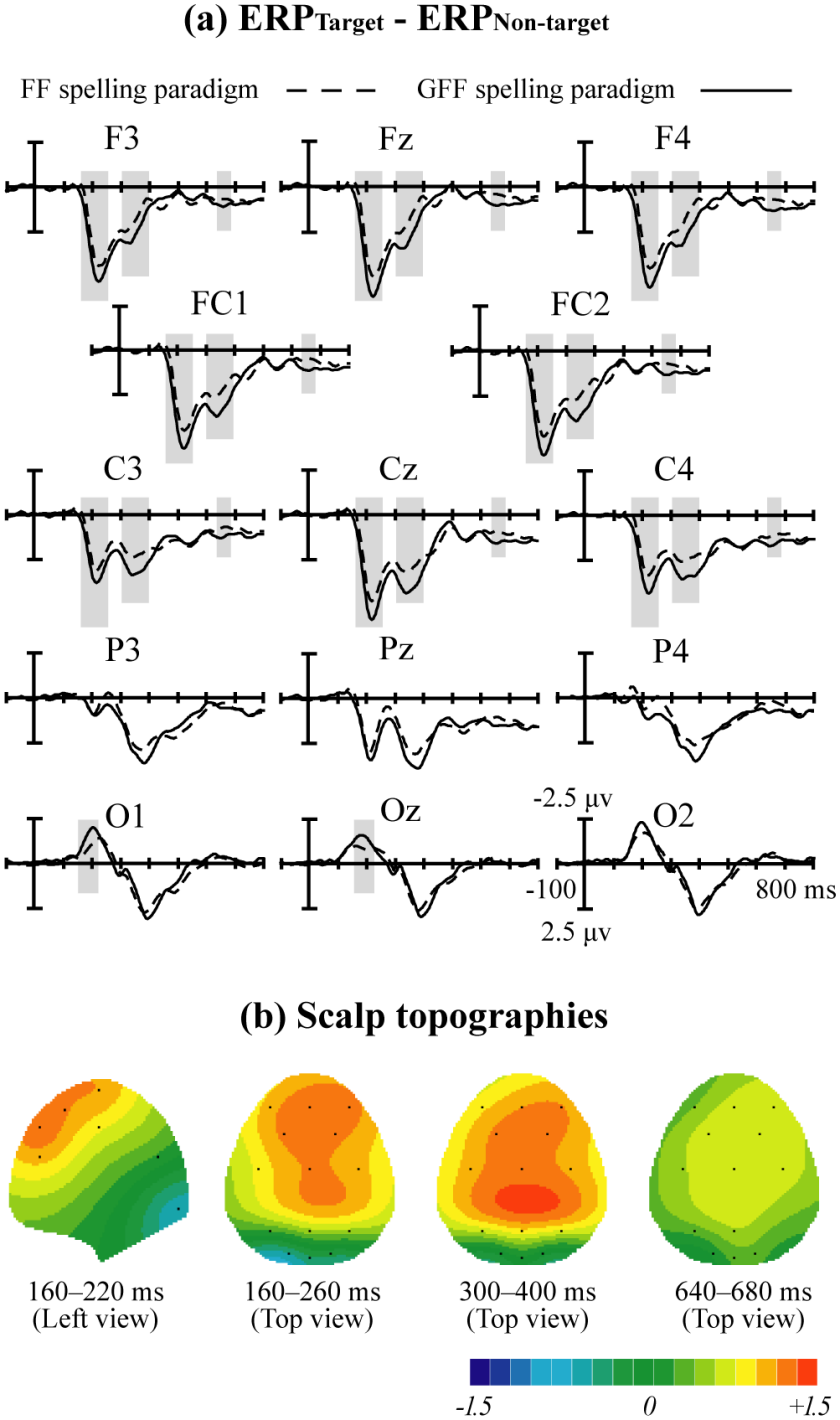
(a) Superimposed difference waveforms of ERPs elicited by the target and non-target trials (ERP_Target_—ERP_Non-target_) in the FF and GFF spelling paradigms. The gray square areas indicate the time periods during which the difference waveforms of ERPs elicited by the target and non-target trials (ERP_Target_—ERP_Non-target_) were significantly different (p < 0.01) between the FF and GFF spelling paradigms. (b) Scalp topographies for double-difference waveforms obtained by subtracting the (ERP_Target_—ERP_Non-target_) waveforms for the FF spelling paradigm from those for the GFF spelling paradigm for the time periods showing significant differences (160–220 ms, 160–260 ms, 300–400 ms, and 640–680 ms).

A greater difference between target and non-target trials would make their classification easier. Therefore, the ERP waveforms elicited by the non-target trials were subtracted from those elicited by the target trials (ERPTarget—ERPNon-target) for the FF and GFF spelling paradigms (Fig 5a). Although the difference waveforms (ERPTarget—ERPNon-target) were similar between the two paradigms, differences could be observed. The statistical differences in the amplitudes measured at 14 electrode sites from 0 to 800 ms during the FF and GFF spelling paradigms were determined using a multiple comparison analysis. Statistically significant differences were found during the following four time periods: (1) 160–220 ms at the left occipital area, (2) 160–260 ms at the frontal-central area, (3) 300–400 ms at the frontal-central area, and (4) from 640–680 ms at the frontal-central area (Fig 5a). The amplitudes of (ERPTarget—ERPNon-target) were higher for the GFF spelling paradigm than for the FF spelling paradigm. Fig 5b depicts the scalp topographies for double-difference waveforms obtained by subtracting the (ERPTarget—ERPNon-target) waveforms in the FF spelling paradigm from those in the GFF spelling paradigm for the four time periods showing significant differences.

**Fig 6:**
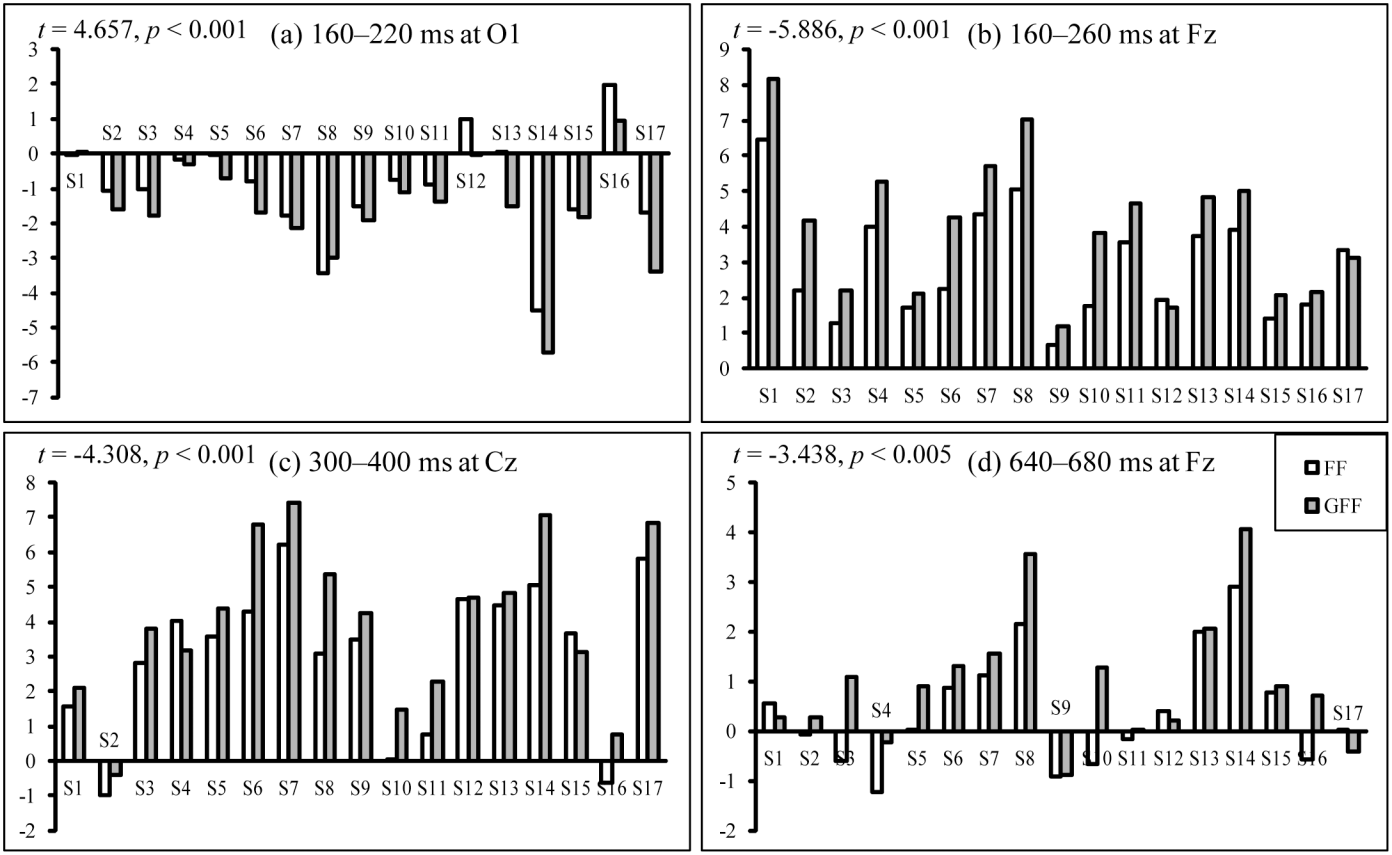
The comparison of averaged amplitudes of (ERP_Target_—ERP_Non-target_) in four significant time periods (160–220 ms at O1, 160–260 ms at Fz, 300–400 ms at Cz, and 640–680 ms at Fz) for 17 individual subjects.

The grand-averaged ERP waveforms in Fig 5 did not adequately reflect the difference between the spelling paradigms within individual subjects. As individual differences are crucial in the BCI system, we compared the averaged amplitudes of (ERPTarget—ERPNon-target) in the four significant time periods (160–220 ms at O1, 160–260 ms at Fz, 300–400 ms at Cz, and 640–680 ms at Fz) for 17 individual subjects (Fig 6). In most subjects, the mean amplitudes in the four significant time periods were significantly larger in the GFF spelling paradigm compared to the FF spelling paradigm.

### Offline classification results

As shown in Fig 5, highly significant differences for the ERPTarget—ERPNon-target waveforms between the GFF and FF spelling paradigms were found during the 160–220 ms, 160–260 ms, 300–400 ms, and 640–680 ms time periods. Therefore, we used the 160–688 ms time window at 14 electrodes as the classification epoch in order to reduce the computational time. First, the original EEG data were filtered between 0.1 and 30 Hz using a third-order Butterworth band pass filter. The EEG was then down-sampled from 250 Hz to 62.5 Hz by selecting every four samples from the epoch. Because we used 14 channels, the size of the feature vector was 14 × 33 (14 channels by 33 time points).

**Fig 7:**
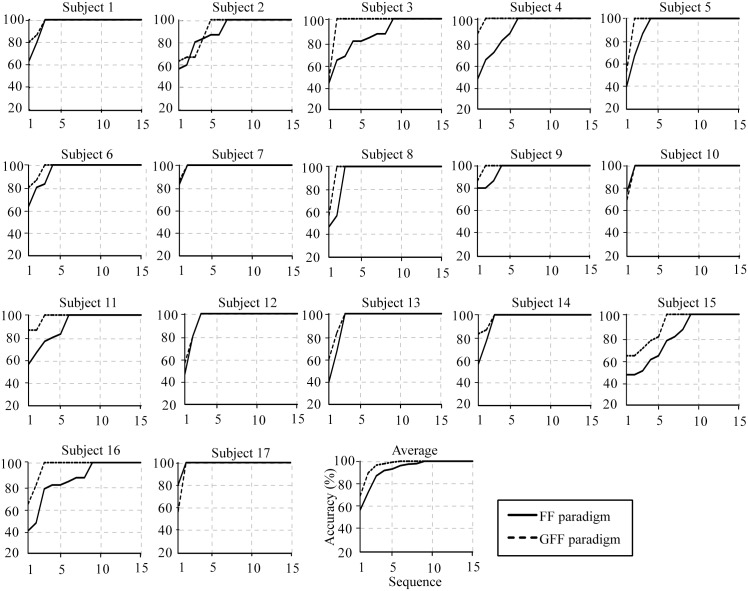
Individual and average accuracies of the P300-speller for 17 subjects in the FF and GFF spelling paradigms.

**Fig 8:**
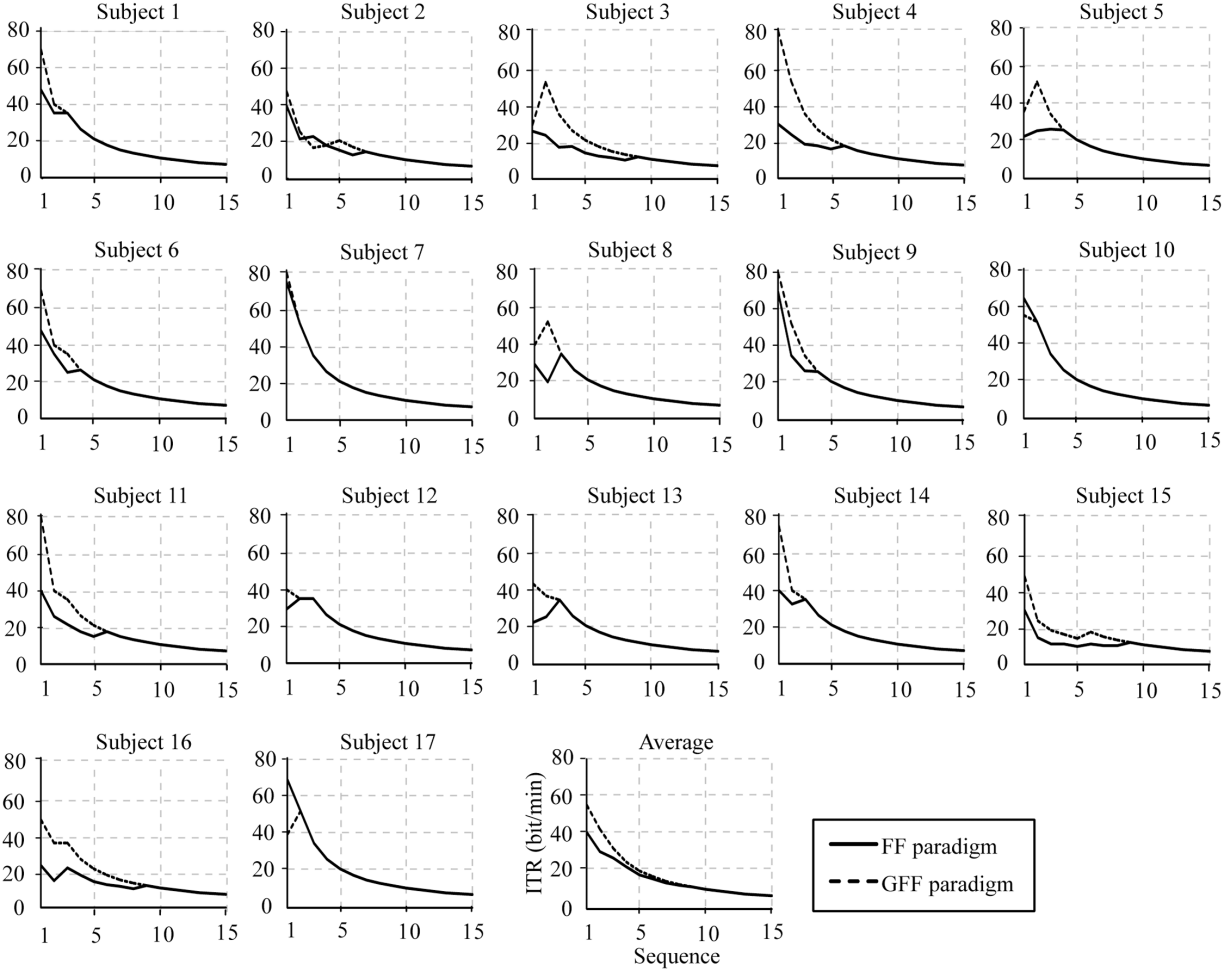
Individual and average ITRs of the P300-speller for 17 subjects in the FF and GFF spelling paradigms.

Results of individual and average accuracies of the P300-speller for 17 subjects in both spelling paradigms are shown in Fig 7. The analysis of the 17 subjects indicated that accuracy increased with sequence number in both paradigms. The average classification accuracy of the P300-speller was greater in the GFF spelling paradigm than in the FF spelling paradigm for 1–9 sequences. Fig 8 illustrates the individual and average ITRs of the P300-speller for 17 subjects in the FF and GFF spelling paradigms. The average ITR of the P300-speller was higher in the GFF spelling paradigm than in the FF spelling paradigm for 1–9 sequences. In addition, we counted the number of sequences needed for subjects to achieve a 100% and ≥70% accuracy level in both spelling paradigms. A level of ≥70% may be regarded as a minimum level of communication [30–31]. Regardless of whether the accuracy level was 100% (p < 0.005) or ≥70% (p < 0.01), the results of the paired t-test indicated that the number of sequences was significantly reduced in the GFF spelling paradigm. The subjects required 2.82 ± 0.27 (mean ± standard deviation) sequences to achieve the goal of 100% classification accuracy in the GFF spelling paradigm, whereas 4.65 ± 0.61 sequences were needed to achieve the same goal in the FF paradigm. A level of ≥70% was achieved with 1.71 ± 0.21 stimulus sequences in the GFF spelling paradigm, whereas the subjects achieved this goal with 2.53 ± 0.31 stimulus sequences in the FF spelling paradigm.

### Online classification results

Table 1 shows the online classification accuracy and ITR for each subject by using two sequences. The best performance, with an accuracy of 96.7% and an ITR of 48.2 bits min-1, was yielded by the GFF paradigm. The results of the paired t-tests showed that the accuracy (p < 0.05) and ITR (p < 0.01) was significantly different between the two spelling paradigms. The mean classification accuracy in the GFF paradigm was 10.5% higher than that of the FF paradigm, while the mean ITR in the GFF paradigm was 7.4 bits min-1 higher than that of the FF paradigm.

**Table 1:** Online classification accuracy and ITR of each subject with two sequences for the FF and GFF spelling paradigms.

	Accuracy (%)		ITR (bit/min)	
Subject	FF paradigm	GFF paradigm	FF paradigm	GFF paradigm
S1	80.0	80.0	34.2	34.2
S2	53.3	86.7	17.6	40
S3	83.3	83.3	36.4	36.4
S4	90.0	93.3	41.9	44.4
S5	80.0	96.7	34.2	48.2
S6	80.0	86.7	34.2	39.4
S7	76.7	83.3	32.1	36.4
S8	50.0	86.7	16.1	39.5
S9	73.3	83.3	29.4	36.4
S10	70.0	73.3	27.4	29.4
S11	80.0	86.7	34.2	39.5
S12	90.0	93.3	41.9	44.4
Avg. ± SD	75.6 ± 12.6	86.1 ± 6.3	31.6 ± 8.1	39.0 ± 5.0
p-value	t = -2.969; p < 0.05		t = -3.139; p < 0.01	

## Discussion

In the present study, we assessed grand-averaged ERP waveforms elicited by target trials in both the FF and GFF spelling paradigms. In addition, we analyzed the difference waveforms of ERPs elicited by the target and non-target trials (ERPTarget—ERPNon-target), and compared the offline and online classification performance of the P300-speller in the FF and proposed GFF spelling paradigms.

In both paradigms, a negative ERP component elicited by the target trials was found at around 150–250 ms at the temporal occipital area (Fig 4). This ERP component is similar to N170, which is involved in face recognition [22, 32–34]. Consistent with a previous study, the mean amplitude of the N170 at the O2 electrode was stronger than that at the O1 electrode, which was attributed to the right hemisphere advantage [21]. In addition, a positive ERP component was observed at around 180–380 ms at the frontal area (Fig 4), which may represent the vertex positive potential (VPP), a potential implicated in face-sensitive brain responses reflecting the neural processing of faces [20]. Another positive ERP component between 300 and 450 ms was found at the parietal area (Fig 4), which may well represent the expected P300 [7].

The performance of the P300-speller system could be improved by enhancing the difference between target trials and non-target trials [19]. Therefore, the (ERPTarget—ERPNon-target) difference waveform was computed by subtracting ERP waveforms elicited by non-target trials from ERP waveforms elicited by target trails in the FF and GFF spelling paradigms (Fig 5a). Our results indicated four statistically significant differences between the two spelling paradigms, and the amplitudes of (ERPTarget—ERPNon-target) in the GFF spelling paradigm were significantly larger than those in the FF spelling paradigm. The first three time periods with significant differences were respectively consistent with those of the N170, VPP, and P300. Previous studies showed that the presentation of novel faces, such as inverted faces, could elicit a larger N170 and VPP, thereby improving the performance of the P300-speller system [19–20]. The green familiar faces shown in the GFF spelling paradigm were more novel for the participants, which increased the amplitude of the N170 and VPP, enabling improved classification relative to the FF spelling paradigm. In addition, many previous studies have reported that different colors are associated with different arousal levels. Compared to low-arousal stimuli, high-arousal stimuli produced larger P300 amplitudes [35–37]. The arousal level associated with green is relatively high [38], which may have accounted for the increased P300 amplitude in the GFF spelling paradigm compared to the FF spelling paradigm. The fourth significant difference with the time period of 640–680 ms was likely to originate from the influence of the P600f, an ERP component demonstrated to be related to the processes involved in the recollection of familiar faces [22, 39]. Our results indicated that the GFF spelling paradigm required more recollection of faces, which led to the amplitude difference in P600f between the two spelling paradigms.

Contrary to previous findings, our results indicated that familiar faces did not elicit the N400f, a strong negative component observed at 300–500 ms post-stimuli [10, 19]. One possible explanation for this finding is that the faces (i.e., David Beckham) were not familiar enough to the participants. A recent study demonstrated that the N400f was best elicited by pictures of family members [23]. In addition, as the N400f overlaps with the P300, the N400f may have also been canceled out by the P300.

Based on the analysis of the ERP components, we selected the 160–688 ms time window as the classification epoch. As expected, both offline and online results indicated that the GFF spelling paradigm obtained significantly higher classification accuracies and ITRs than the FF spelling paradigm. The offline and online results testified that the GFF spelling paradigm induced larger ERP components, resulting in the improvement of P300-speller performance.

Generally, ITR is used as an important statistical metric for a BCI system [4, 20, 29]. The ITR depends on both classification accuracy and speed of character selection. Moreover, the speed is based on the number of stimulus sequence used for averaging and ISI [15, 40]. The reduction in the number of stimulus sequences could shorten the speed of character selection, but this reduction inevitably decreases the signal-to-noise ratio and thus typically entails a decrease in classification accuracy. Our individual results indicated that the ITRs were maximal in some subjects when the stimulus sequence was repeated twice. In addition, decreasing ISI would result in less time for character selection, but a decrease in ISI leads to smaller P300 amplitudes and larger latencies, which would decrease classification accuracy. Therefore, classification accuracy and speed of character selection must be weighed for obtaining higher ITR in the design of the BCI system. A recent study reported that different spelling paradigms might require different ISIs [41]. Future studies should examine further the effects of ISI in the GFF spelling paradigm.

## Conclusion

This study investigated whether the GFF spelling paradigm would lead to better P300-speller performance compared to the FF spelling paradigm. Our results indicated a highly significant improvement in the GFF spelling paradigm. This optimization may have a significant impact on increasing the communication speed and accuracy of the P300-speller. The performance of the P300-speller does not depend solely on one attribute; rather, multiple factors are at play. Therefore, further research is necessary to determine the influence of multiple attributes on P300-speller performance.
